# Investigating the Impact of Superabsorbent Polymer Sizes on Absorption and Cement Paste Rheology

**DOI:** 10.3390/ma17133115

**Published:** 2024-06-25

**Authors:** Nilam Adsul, Jun-Woo Lee, Su-Tae Kang

**Affiliations:** 1 Department of Civil Engineering, Daegu University, Gyeongsan 38453, Republic of Korea; adsulnil@daegu.ac.kr (N.A.); dlwnsdn518@naver.com (J.-W.L.); 2 Department of Architecture Engineering, Daegu University, Gyeongsan 38453, Republic of Korea

**Keywords:** cement paste, water-to-cement ratio, superabsorbent polymer, absorption capacity, plastic viscosity, yield stress

## Abstract

This study aims to understand the water retention capabilities of Superabsorbent Polymers (SAPs) in different alkaline environments for internal curing and to assess their impact on the rheological properties of cement paste. Therefore, the focus of this paper is on the absorption capacities of two different sizes of polyacrylic-based Superabsorbent Polymers : SAP A, with an average size of 28 µm, and SAP B, with an average size of 80 µm, in various solutions, such as pH 7, pH 11, pH 13, and cement filtrate solution (pH 13.73). Additionally, the study investigates the rheological properties of SAP-modified cement pastes, considering three different water-to-cement (*w*/*c*) ratios (0.4, 0.5, and 0.6) and four different dosages of SAPs (0.2%, 0.3%, 0.4%, and 0.5% by weight of cement). The results showed that the absorption capacity of SAP A was higher in all solutions compared to SAP B. However, both SAPs exhibited lower absorption capacity and early desorption in the cement filtrate solution. In contrast to the absorption results in pH 13 and cement filtrate solutions, the rheological properties, including plastic viscosity and yield stress, of the cement paste with a *w*/*c* ratio of 0.4 and 0.5, as well as both dry and wet (presoaked) SAPs, were higher than those of the cement paste without SAP, indicating continuous absorption by SAP. The viscosity and yield stress increased over time with increasing SAP dosage. However, in the mixes with a *w*/*c* ratio of 0.6, the values of plastic viscosity and yield stress were initially lower for the mixes with dry SAPs compared to the reference mix. Additionally, cement pastes containing wet SAP showed higher viscosity and yield stress compared to the pastes containing dry SAP.

## 1. Introduction

Superabsorbent polymers (SAPs) are highly versatile materials renowned for their remarkable liquid absorption capabilities, allowing them to absorb liquid volumes many times their weight. These properties make them widely applicable in diverse industrial sectors, including agriculture, healthcare, effluent treatment, and civil construction [1]. The integration of SAP into cementitious materials has been studied extensively, such as admixtures, internal curing agents, shrinkage reducers, self-healing agents, and freeze and thaw resistance enhancers [2,3]. Many researchers have investigated the incorporation of SAPs in different types of concrete, such as high-performance concrete, high-strength concrete, self-compacting concrete, ultra-high-performance concrete, and alkali-activated slag mortar, in combination with various supplementary cementitious materials [4,5,6,7,8].

Water absorption into SAPs occurs due to osmotic pressure, which expands the space between polymer chains and cross-links. This osmotic pressure is driven by the concentration gradient of mobile ions between the gel and the solution. Therefore, the presence of dissolved ions such as K^+^, Na^+^, Mg^2+^, and Ca^2+^ in the surrounding solution affects the osmotic pressure and, thus, influences the swelling capacity of SAPs [2,9,10].

The absorption capacity and rate of SAPs can be modified based on chemical compositions, physical attributes, and the properties of the surrounding fluid, including composition, ionic concentration, pH, temperature, and pressure [9,11]. Additionally, the particle size, quantity, and chemical composition of SAPs play a crucial role in their performance in concrete, impacting mechanical stability during mixing and rheological properties such as yield stress, plastic viscosity, and thixotropy over time [12,13,14,15]. Likewise, the type and properties of SAPs influence workability and setting times [16,17].

Different types and sizes of SAPs have varying effects on fresh concrete properties because of their different absorption characteristics. For instance, incorporating acrylamide/acrylic sodium copolymer SAP (with mean sizes of 471.3 and 95.1 µm) with additional water initially reduces yield stress, followed by an increase over time, accompanied by a simultaneous reduction in plastic viscosity. In contrast, incorporating acrylamide-based SAP (with a mean size of 63 µm) with extra water leads to a continuous rise in yield stress and plastic viscosity over time as the dosage of SAP increases, owing to its lack of desorption properties [12]. In a study by Ma et al. (2019), acrylamide/acrylic acid copolymers with average diameters of 125 µm and 840 µm were utilized. The mortar with the larger SAP particle size exhibited higher maximum shear stress, plastic viscosity, and relative thixotropy compared to the mortar with the smaller SAP size [18]. On the other hand, Lee et al. (2018) studied two types of SAPs, where polyacrylate-based SAP exhibited a lower swelling ratio and slower swelling recovery compared to polyacrylate-co-acrylamide-based SAP, as it is more prone to the calcium ions present in the solution [19].

The absorption properties of SAPs are influenced by the *w/c* ratio, superplasticizer, and supplementary cementitious materials, subsequently affecting the rheological properties of cementitious materials with SAP [13]. Higher *w/c* ratios lead to more excellent SAP absorption, causing more significant changes in Bingham parameters for mixes with SAP [10,13,20]. As SAP content increases, more dosage of water-reducing agent is needed to maintain workability. However, excess SAP content without additional water or superplasticizer reduces the workability of the cementitious material [20,21]. The method of SAP addition, whether presoaked or dry, significantly affects slump more than particle size. Concrete with presoaked SAP with additional water showed an increased slump, while concrete with dry SAP exhibited a decreased slump with an increased SAP dosage [22,23].

However, certain aspects of the performance of different sizes of SAP, especially very fine particle sizes, in varying dosages without additional water within a cementitious environment, particularly regarding rheological properties, require further investigation. This will help determine the effect of SAP alone in cement paste and identify the suitable dosage to enhance the properties of cement paste.

This study investigates the absorption and desorption characteristics of SAPs in different pH and cement filtrate solutions. The aim is to understand how long SAP can absorb solutions and when it will start the desorption process in various solution mediums. Additionally, this study explores the impact of two different sizes of SAPs as internal curing agents on the plastic viscosity and yield stress of cement paste. The cement paste was prepared with varying dosages of SAPs (0.2% to 0.5%) and three different *w*/*c* ratios (0.4 to 0.6). Furthermore, the study examines the influence of both dry and wet SAP on the rheological properties. The results of the absorption and desorption by SAP in different solutions will contribute to understanding the changes in viscosity and yield stress of cement paste with SAP over time.

## 2. Materials and Methods

### 2.1. Material and Sample Preparation

**Figure 1:**
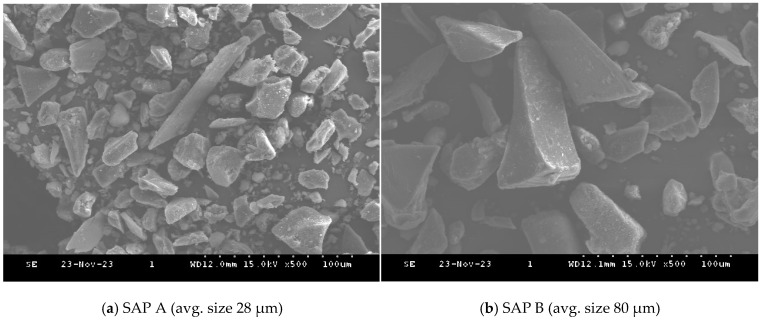
SEM images of the particle sizes of SAP A and SAP B.

In this study, Ordinary Portland Cement (Type I, Hanil cement, Seoul, Republic of Korea) and Polyacrylic-based SAP from TPY Co. Ltd., Hwaseong-si, Republic of Korea, in two different sizes: SAP A (avg. size 28 µm-TPY 900) and SAP B (avg. size 80 µm-TPY 502) were utilized. The scanning Electron Microscope (SEM) (S-4300, Hitachi Ltd., Tokyo, Japan) images of the particle size of both SAPs are depicted in Figure 1. The cement paste was prepared using three different water-to-cement ratios (0.4, 0.5 and 0.6) and varying dosages of SAP (0.2%, 0.3%, 0.4% and 0.5% by weight of cement) as outlined in Table 1 and tested for rheological properties. The addition of SAP was carried out in two different ways: dry (D) and wet (W), with SAP A denoted as A and SAP B as B. During experimental work, it was noticed that a *w*/*c* ratio of less than 0.4 resulted in a highly viscous mix, which was not suitable for rheology testing. Therefore, the *w*/*c* ratios were selected above 0.4. 

Throughout mix preparation and rheology testing, room conditions were maintained at 20 ± 2 °C and 30 ± 5% Relative Humidity (RH). The water used for cement paste preparation was consistently kept at 20 °C using a water bath (Joanlab Equipment Co., Ltd., Huzhou, China).

The mixing procedure for all cement pastes used in the present study is as follows:

(a) Dry SAP mixing in cement paste:
  Initially, all dry materials, such as cement and dry SAP, were mixed at speed 2 (95 rpm) using a Hobart mixer for 60 s. Afterward, water was added to the dry materials and mixed at speed 2 (95 rpm) for 30 s. Then, the mixer was stopped, and the cement paste was scraped from the walls and bottom of the mixing bowl (50 s). After that, the cement paste was mixed at speed 2 (95 rpm), and the bottom of the mixing bowl was scraped again (40 s). Finally, the paste was mixed at speed 4 (135 rpm) for 60 s.

(b) Wet SAP mixing in cement paste:
  The dry SAP and water were mixed and left to presoak for 30 min. The dry cement alone was mixed at speed 2 (95 rpm) to remove lumps (if any) for 60 s. After presoaking the SAP, the SAP and water mixture was added to the cement and mixed at speed 2 (95 rpm) for 60 s. Then, the mixer was stopped, and the cement paste was scraped from the walls and bottom of the mixing bowl for 30 s. Furthermore, the paste was mixed at speed 2 (95 rpm) and again the bottom of the bowl was scraped (60 s). Finally, the paste was mixed at speed 4 (135 rpm) for 60 s.

### 2.2. Testing Methods

#### 2.2.1. Absorption Capacity of SAP

Initially, SAP absorption was studied in three different buffer solutions—pH 7, pH 11, and pH 13—to investigate the impact of pH on its absorption. The pH 7 represented water, pH 11 represented a mildly alkaline concrete admixture environment, and pH 13 represented a highly alkaline cementitious environment.

Additionally, absorption capacity was tested in a cement filtrate solution. The cement filtrate solution was prepared using cement and deionized water with a *w*/*c* ratio of 5. The cement and water paste were continuously stirred using a mixer for 24 h at a constant speed, and after that, the liquid was filtered.

**Figure 2:**
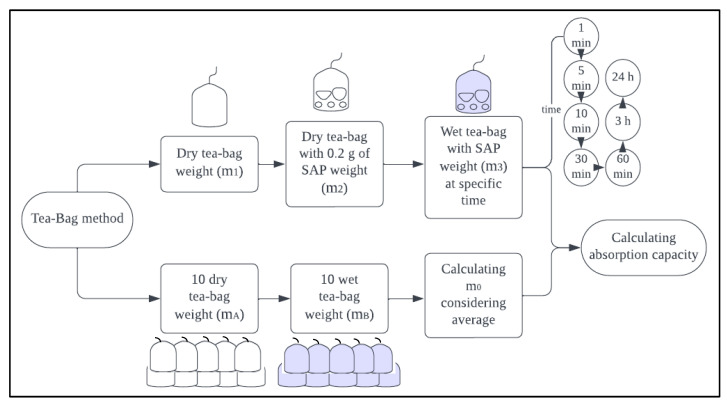
Tea-bag method.

**Figure 3:**
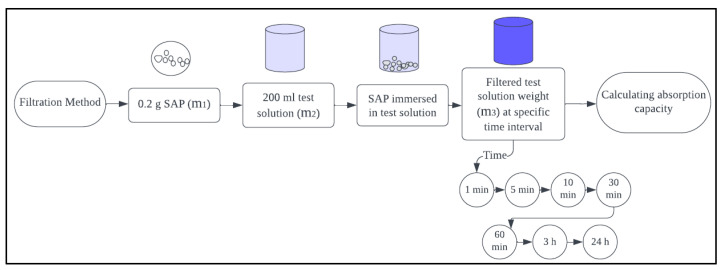
Filtration method.

Furthermore, following RILEM guidelines [24,25], two testing methods—the ‘tea-bag method’ and the ‘filtration Method’—were used to investigate the absorption capacity of SAP, as shown in Figure 2 and Figure 3, respectively. In the tea-bag method, a dry tea bag was initially weighed as mass $m_{1}$, and a dry tea bag containing 0.2 g of SAP was weighed as mass $m_{2}$. The tea bag was then sealed to prevent any dry or swollen SAP from leaking out. Further, the SAP-filled tea bag was soaked in a beaker filled with the test solution (about 200 mL). During this process, the beaker was covered with a thin plastic film to prevent carbonation and evaporation. The soaked SAP-filled tea bag was removed at specific time intervals: 1, 5, 10, 30, 60 min, 3 h and 24 h after the SAP/liquid contact time. The tea bag was wrapped with a dry tissue, and a weight of 1 kg was placed on it for 60 s to remove surface water before measuring the weight. This was performed to avoid errors caused by manual pressure applied by hand while removing surface water from the tea bag [26]. Afterwards, the weight $m_{3}$ was recorded. Furthermore, $m_{0}$ was calculated by taking into consideration the average dry and wet weight of the tea bag as shown in Equation (1), where *n* is the number of tea bags used (total of 10 tea bags), $m_{Ai}$ and $m_{Bi}$ are the individual masses of the dry and wet tea bags, respectively. Consequently, the absorption capacity was calculated according to Equation (2).

(1) $$m_{0}=\frac{1}{n}\sum_{i=1}^{n}\left(m_{Bi}-m_{Ai}\right)$$

(2) $$\mathrm{Absorption}\;\mathrm{capacity}\;\mathrm{by}\;\mathrm{tea}\text{-}\mathrm{bag}\;\mathrm{method}=\frac{m_{3}-m_{2}-m_{0}}{m_{2}-m_{1}}$$

In the filtration method, the dry amount of 0.2 g of SAP was measured as mass $m_{1}$, and the test solution of 200 mL was measured as mass $m_{2}$. The test fluid was added to a beaker, and dry SAP was immersed. Immediately, the beaker was tightly sealed using a thin plastic film to prevent evaporation and carbonation. The entire solution was filtered after time intervals of 1, 5, 10, 30, 60 min, 3 h and 24 h from the SAP/liquid contact time, and the amount of filtered fluid was determined as mass $m_{3}$. The filter paper (12–15 µm mesh size) used for filtering was saturated in the test solution before use, and the filtration was carried out using a funnel with filter paper. Different containers with new solutions and SAP were prepared for each testing interval. Equation (3) was used to calculate absorption capacity by filtration method.

(3) $$\mathrm{Absorption}\;\mathrm{capacity}\;\mathrm{by}\;\mathrm{filtration}\;\mathrm{method}=\frac{m_{2}\;–\;m_{3}\;}{\;m_{1}}$$

#### 2.2.2. Rheological Testing

Cement pastes, immediately after mixing, were poured into a cylinder, and rheological tests using a Brookfield DV2T rheometer (Brookfield AMETEK, Middleborough, MA, USA) were conducted. The rheometer was connected to a PC, and data were recorded in RheocalcT software (https://www.brookfieldengineering.com/products/software/rheocalct, accessed from 14 January 2024). During testing, the cylinder was covered with thin plastic to prevent evaporation. In this study, the Bingham model was adopted, which includes yield stress and viscosity, as shown in Equation (4),

(4) $$\tau =\tau_{0}+µ\;\gamma$$

where τ is the shear stress, $\tau_{0}$ is the yield stress, *µ* is the plastic viscosity of the cementitious suspensions, and *γ* is the shear strain rate.

**Figure 4:**
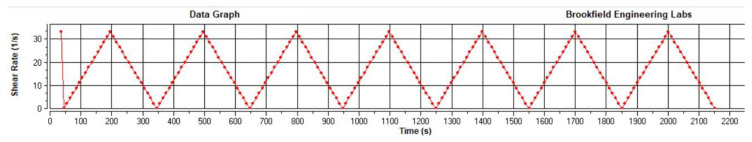
Shear rate applied during the test duration.

The torque required to rotate the spindle and the shear rate were determined. The samples were pre-sheared for 30 s at 33.15 s⁻^1^, followed by a 10 s rest. Then, the shear rate was ramped up from 0 to 33.15 s^−1^ and down from 33.15 to 0 s^−1^, which was repeated throughout the test duration of about 35 min, as shown in Figure 4. The shear rate at each step was maintained for 10 s to measure a stable shear stress. The curves obtained during the decreasing rate were more consistent. Furthermore, a linear regression was performed on the experimental data, plotting shear stress against shear rate. The slope and intercept of the plotted regression line were used to determine the plastic viscosity and yield stress [27]. The evaluations were conducted at 5, 10, 15, 20, 25, 30, and 35 min.

## 3. Results and Discussion

### 3.1. Absorption Capacities

The absorption capacities of two sizes of SAPs, namely SAP A, with an avg. size of 28 µm, and SAP B, with an avg. size of 80 µm, were studied in different solutions (pH 7, pH 11, pH 13, and cement filtrate solution with a pH of 13.73) using both the tea-bag and filtration methods. These tests were conducted at intervals of 1, 5, 10, 30, 60 min, 3 h, and 24 h.

**Figure 5:**
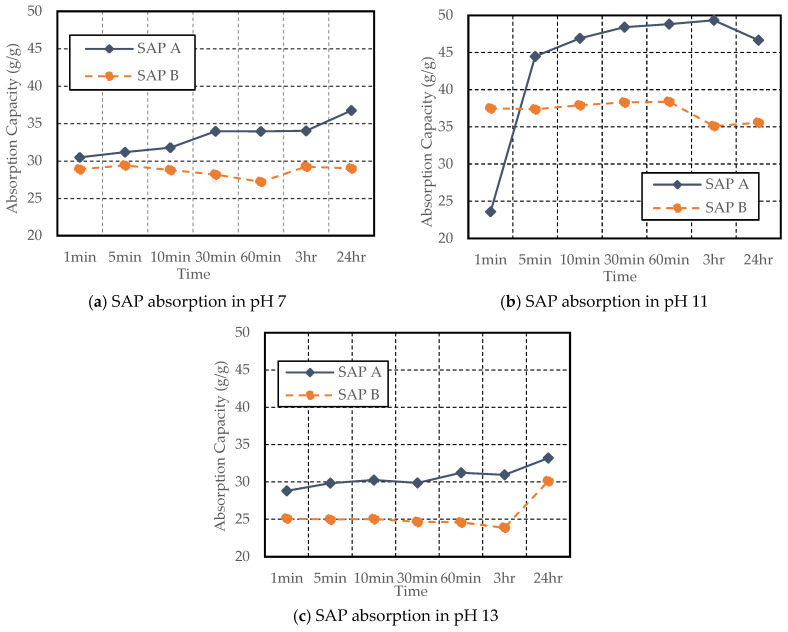
SAP absorption testing using the tea-bag method in pH 7, pH 11, and pH 13 solution.

**Figure 6:**
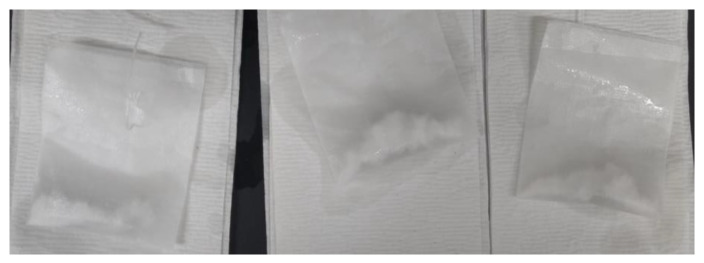
Gel blocking observed in the tea-bag method by SAP A in the first few minutes.

From the results of the tea-bag method presented in Figure 5, it is evident that SAP A exhibited a higher absorption capacity than SAP B despite having a smaller particle size. Likewise, a study by Kazemian and Shafei (2024) revealed that the fine-sized SAP exhibited a 10% higher absorption capacity when compared with the large SAP particles [28]. The smaller size of SAP A led to a delayed initial absorption, attributed to gel-blocking, as illustrated in Figure 6. Gel blocking is a characteristic of very fine SAP particles smaller than 100 µm. In this scenario, minimal absorption occurs on the surface when SAP encounters liquid, causing the slightly swollen particles to adhere to each other, forming clusters that contain a significant amount of unswollen SAP, hindering further disaggregation and absorption [2,29,30].

SAP’s initial absorption capacity was lower in all pH solutions; however, significantly lower initial absorption was noted in the pH 11 solution, as shown in Figure 5b. The absorption test was conducted twice to confirm the changes in SAP absorption in the pH 11 solution; however, it showed a similar trend each time. This may be due to small particles aggregating and swelling, leading to gel blocking. Over time, SAP A’s absorption capacity increased from 23.59 g/g at 1 min to 49.35 g/g at 3 h in pH 11. Furthermore, SAP A demonstrated an increase in absorptivity in all three pH solutions, with the highest absorption in pH 11 (46.65 g/g at 24 h) compared to pH 7 (36.74 g/g at 24 h) and pH 13 (33.20 g/g at 24 h).

In contrast, SAP B exhibited signs of water release in all cases after 30–60 min. However, in pH 13, absorption capacity was significantly increased from 23.89 g/g at 3 h to 30.10 g/g at 24 h. The increase in pH from 11 to 13 resulted in a decrease in absorption capacity for both SAPs, consistent with studies conducted by [11,26].

**Figure 7:**
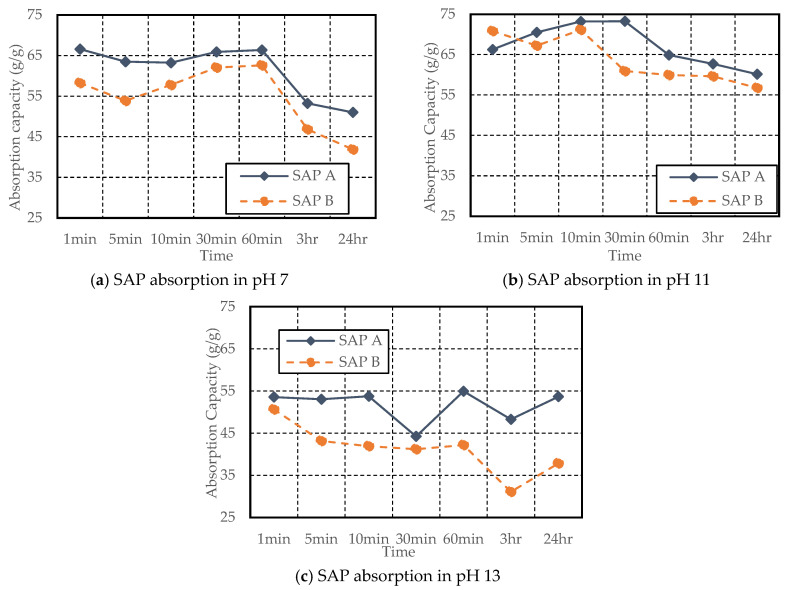
SAP absorption testing using filtration method in pH 7, pH 11, and pH 13 solution.

Both SAPs showed higher absorptivity in the filtration method than the tea-bag method [25,31]. Like the tea-bag method, SAP A’s absorption capacity was higher in all three pH solutions than SAP B’s, as shown in Figure 7. In the pH 7 solution, both SAPs initially exhibited increased absorption, followed by a significant decreasing trend after 60 min. In pH 11, SAP A showed lower early absorption (66.27 g/g at 1 min of contact with solution) than SAP B (70.84 g/g at 1 min). Over time, a reduction in absorption capacity in pH 11 solution was observed after 30 min by SAP A and after 10 min by SAP B. Furthermore, in the filtration method, the absorption by both SAPs was lower in pH 13 compared to pH 7 and pH 11, consistent with a study by [32]. In pH 13, SAP A demonstrated a fluctuating trend, reaching its peak at 54.93 g/g at 60 min, while SAP B exhibited varying capacities, with a minimum of 31.09 g/g at the 3 h mark and generally lower values compared to SAP A across the testing periods, including a notable increase for both SAPs at 24 h.

**Figure 8:**
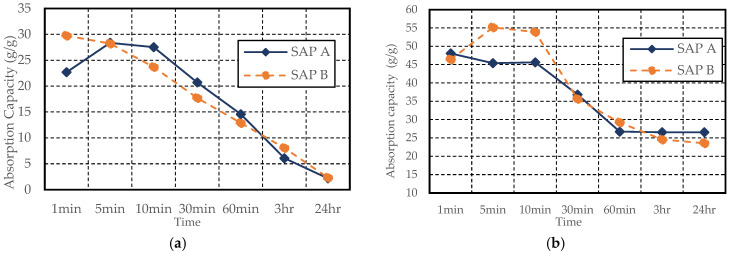
SAP absorption testing in cement filtrate solution (pH 13.73) (**a**) tea-bag method (**b**) filtration method.

**Figure 9:**
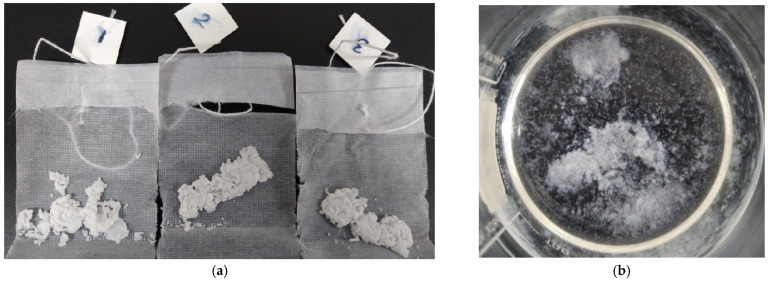
Formation of white egg shell-like crust during (**a**) tea-bag and (**b**) filtration methods in Cement Filtrate.

Both SAPs demonstrated lower absorption in the cement filtrate solution with a higher pH of 13.73, as shown in Figure 8, similar to the findings in [33]. This may be due to the dissolved ions (K^+^, Na^+^, Mg^2+^, and Ca^2+^) in the cement filtrate solution affecting the osmotic pressure and hence influencing the swelling capacity of SAP [2,10]. SAP A and SAP B also formed a white egg-shell-like crust in the cement filtrate solution during both tests. This phenomenon may be attributed to the exposure of SAP to a highly alkaline fluid with an elevated ion concentration, particularly calcium ions [25,34], as shown in Figure 9. The decreasing trend in the absorptivity of polyacrylic-based SAP, as shown in Figure 8, closely resembles the absorptivity observed by Zhong et al. (2020) [31] using acrylic acid and acrylamide-based SAP in the cement filtrate solution in both the test methods. Similarly, in a study by Yun et al. (2017), sodium-polyacrylate-based SAP [35] exhibited a trend similar to that observed in this study. The absorption by SAP A using the tea-bag and filtration methods at 1 min was 22.68 g/g and 48.06 g/g, respectively, which then decreased to 2.19 g/g and 26.54 g/g after 24 h. Similarly, for SAP B, the absorption capacity using the tea-bag and filtration methods at 1 min was 29.74 g/g and 46.39 g/g, respectively, which then decreased to 2.31 g/g and 23.55 g/g after 24 h, respectively.

### 3.2. Rheological Properties

**Figure 10:**
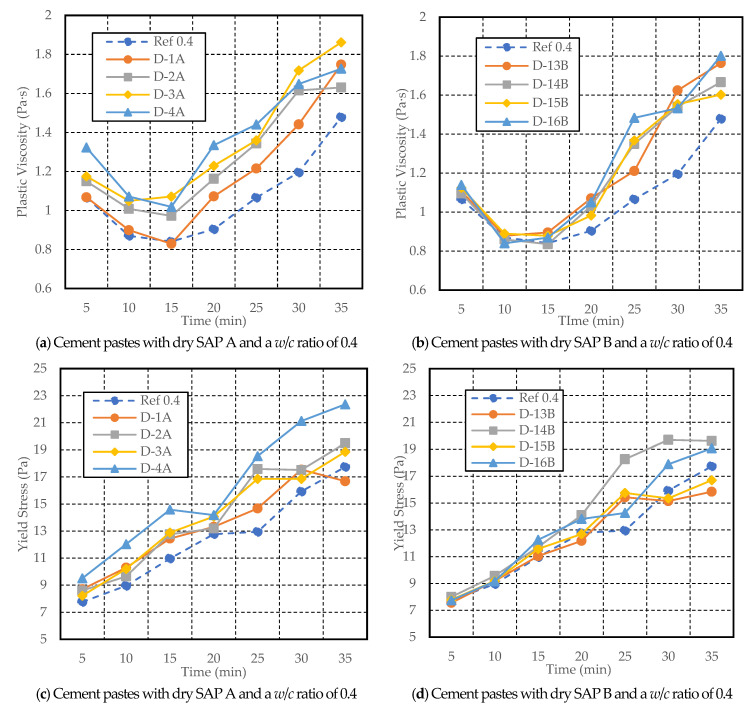
Variations in yield stress and plastic viscosity with time for cement paste with a *w*/*c* ratio of 0.4 and different dosages of dry SAP A and B.

**Figure 11:**
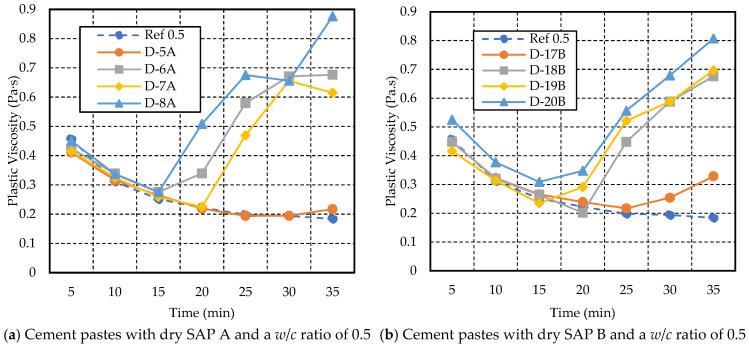

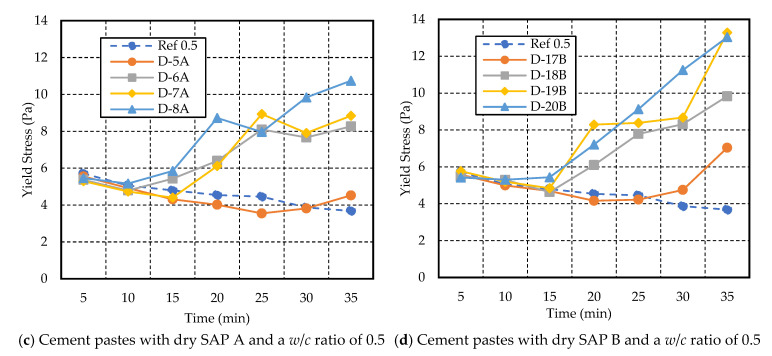
Variations in yield stress and plastic viscosity with time for cement paste with a *w*/*c* ratio of 0.5 and different dosages of dry SAP A and B.

**Figure 12:**
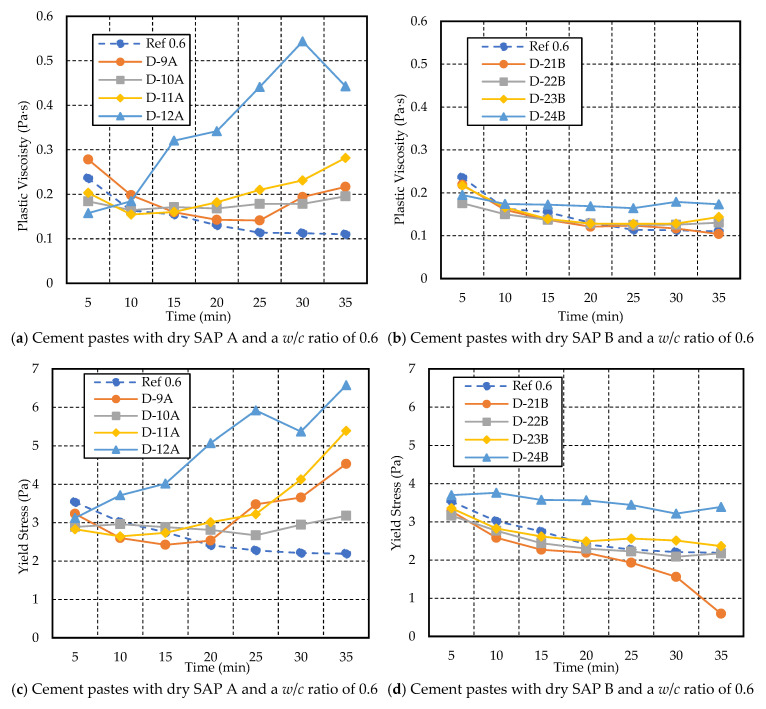
Variations in yield stress and plastic viscosity with time for cement paste with a *w/c* ratio of 0.6 and different dosages of SAP A and B.

**Figure 13:**
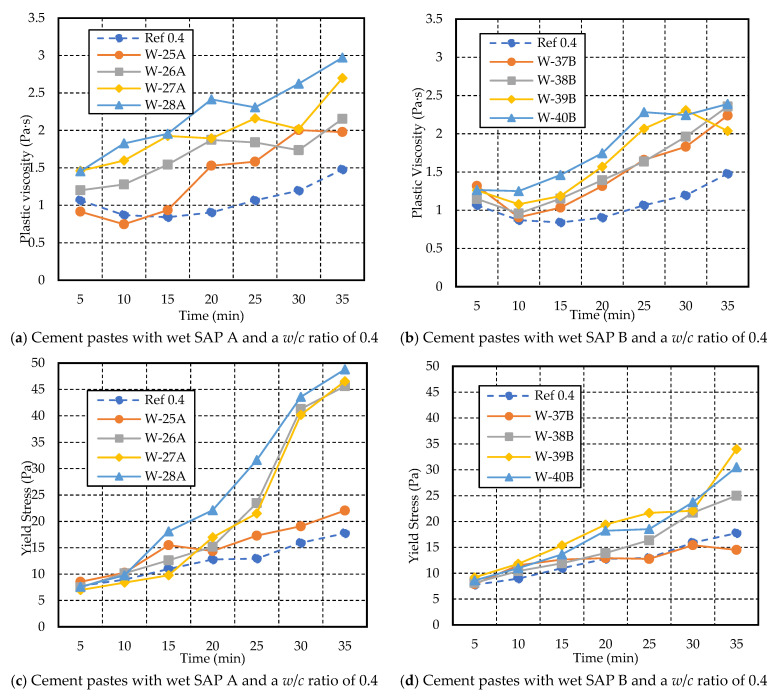
Variations in yield stress and plastic viscosity with time for cement paste with a *w*/*c* ratio of 0.4 and different dosages of wet SAP A and B.

**Figure 14:**
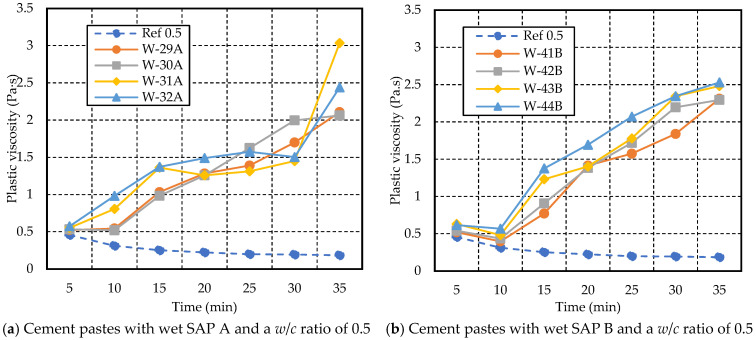

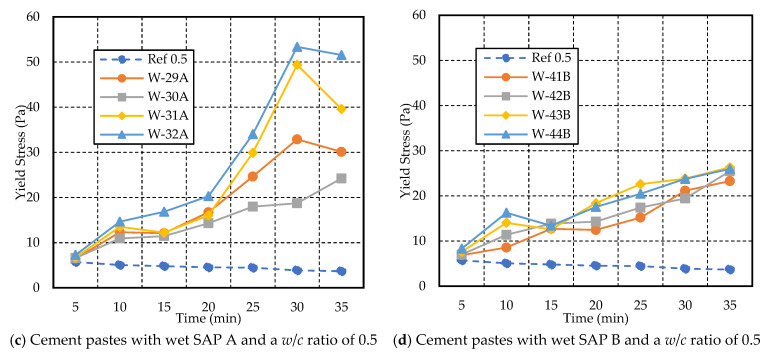
Variations in yield stress and plastic viscosity with time for cement paste with a *w*/*c* ratio of 0.5 and different dosages of wet SAP A and B.

**Figure 15:**
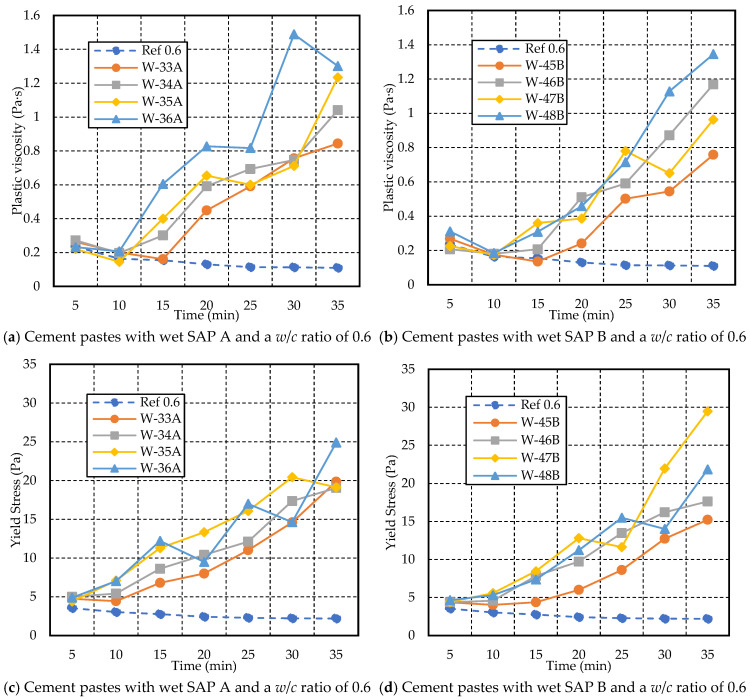
Variations in yield stress and plastic viscosity with time for cement paste with a *w/c* ratio of 0.6 and different dosages of wet SAP A and B.

Figure 10, Figure 11, Figure 12, Figure 13, Figure 14 and Figure 15 illustrate the variations over time in plastic viscosity and yield stress for cement paste with varying dry SAP A and SAP B content (ranging from 0.2 to 0.5% weight of cement) and three different *w*/*c* ratios (0.4, 0.5, and 0.6). Despite the observed desorption behavior of SAP in the cement filtrate solution, the introduction of SAP led to elevated plastic viscosity and yield stress [12,36]. Both SAPs exhibited higher values of plastic viscosity and yield stress in mixes with *w*/*c* ratios of 0.4, 0.5, and 0.6, containing different dosages of dry and wet SAP compared to reference mixes.

Examining Figure 10a,b for cement paste with a *w/c* ratio of 0.4, it is evident that the plastic viscosity increased with the dosage of SAP A and SAP B from 0.2% to 0.5%, compared to the reference mixes. Similar behavior was observed in a study carried out by [37]. However, after 35 min, a reduction in viscosity was noted for D-4A (1.73 Pa·s) compared to D-1A (1.75 Pa·s) and D-3A (1.86 Pa·s) mixes. Whereas, cement paste with a lower content of SAP B (D-13B) showed higher viscosity (1.76 Pa·s) at 30 min than mixes with a higher SAP dosage, i.e., D-14B (1.67 Pa·s) and D-15B (1.60 Pa·s).

The yield stress of 0.3% and 0.4% SAP A (D-2A and D-3A) showed nearly similar trends and mixes with 0.2% SAP B (D-13B) and 0.4% SAP B (D-15B) also exhibited almost similar behavior throughout the test duration as shown in Figure 10c,d. Reduction in yield stress was observed for D-1A from 17.55 Pa to 16.69 Pa (30 to 35 min) compared to the reference mix from 15.93 to 17.74 Pa. Similarly, the yield stress reduced for D-13B from 15.43 Pa (at 25 min) to 15.14 Pa (at 30 min), with a sudden increase by 15.85 Pa (at 35 min), which was lower than the reference mix. The D-15B mix rose from 15.35 Pa at 30 min to 16.70 Pa at 35 min, which was also lower than the reference mix. Meanwhile, D-14B showed a drastic increase in yield stress from 14.09 Pa at 20 min to 19.62 Pa at 35 min.

For cement paste with a *w*/*c* ratio of 0.5 (Figure 11), mixes with 0.3% to 0.5% of dry SAP A and B exhibited higher plastic viscosity and yield stress compared to the reference and 0.2% SAP mix. This aligns with the findings of Oh and Choi (2023) [38]. The viscosity of paste with 0.2% of dry SAP A (D-5A) and the reference showed minimal variation, except for a slight increase observed after 30 min, from 0.19 Pa·s to 0.22 Pa·s. On the other hand, the paste with 0.2% SAP B (D-17B) showed a higher increase in plastic viscosity, reaching 0.45 Pa·s at 5 min, followed by a decreasing trend, with a slight increase observed between 20 min and 35 min, ranging from 0.24 Pa·s to 0.33 Pa·s, compared to the reference mix, which ranged from 0.22 Pa·s to 0.18 Pa·s. The mix containing 0.3% SAP A (D-6A) showed higher viscosity than the 0.4% SAP A (D-7A).

The yield stress of the paste with 0.2% SAP A and B was lower than the reference mix from 5 min to 25 min, after which an increase was noted, as shown in Figure 11c,d. A higher dosage of SAP A (D-8A) and B (D-20B) led to an increase in yield stress. At 35 min, it was the highest, i.e., 10.74 Pa and 13.04 Pa, respectively, compared to the reference mix, which was 3.68 Pa.

Figure 12 illustrates the plastic viscosity and yield stress for cement paste with a *w*/*c* ratio of 0.6. Increasing the dosage of SAP A and SAP B resulted in an increase in plastic viscosity and yield stress over time, closely aligning with the findings of Senff et al. (2015) [39]. However, the mix containing a higher dosage of SAP A (D-12A) showed a drastic increase in plastic viscosity and yield stress over time compared to the other mixes. D-9A, containing 0.2% SAP, initially showed an increase in plastic viscosity, followed by a slight decrease. On the other hand, D-21B exhibited the opposite behavior, experiencing a decrease in yield stress throughout the test duration. When compared, the rheological properties of cement paste with a *w/c* ratio of 0.6 are significantly influenced by SAP A rather than SAP B.

At 30 min, the yield stress of the paste with 0.2% SAP A (D-9A) showed an increase to 4.53 Pa, compared to 3.18 Pa for D-10A and 2.19 Pa for Ref-0.6. Furthermore, higher dosages of both SAP A and B showed higher yield stress compared to the rest of the mixes, which is consistent with the findings in [39].

Based on the above results, a common observation was noted, which was lower initial plastic viscosity and yield stress in the mixes containing SAP. This can be attributed to SAPs absorbing Ca^2+^ and releasing Na^+^ and K^+^ ions into the pore solution. Ca^2+^ initially binds with SAP, thereby reducing the initial swelling. However, over time, the bound Ca^2+^ is displaced, allowing the swelling to gradually recover. Higher alkalinity tends to increase swelling, whereas it decreases with increased calcium concentration. The greater the degree of ion exchange, the lower the swelling of SAP [19].

The cement pastes with a *w*/*c* of 0.4 and wet SAPs (presoaked for 30 min) showed higher plastic viscosity and yield stress with an increase in the dosage of SAP compared to the reference mixes and cement pastes with dry SAP, as shown in Figure 13. However, adding SAP A resulted in greater viscosity when compared to mixes with SAP B. The higher dosages of SAP A (W-28A) and SAP B (W-40B) showed an increase in viscosity, specifically 2.97 Pa·s and 2.39 Pa·s, respectively, in comparison to the viscosity of the Ref-0.4 mix, which measured 1.48 Pa·s at the end of the test duration. Furthermore, W-37B exhibited an increase in plastic viscosity from 1.32 Pa·s (at 20 min) to 2.24 Pa·s (at 35 min), which was quite close to W-38B, where it increased from 1.39 Pa·s (at 20 min) to 2.36 Pa·s (at 35 min).

Regarding yield stress, W-25A increased with time, while W-37B showed a decrease, as shown in Figure 13c,d, compared to Ref 0.4. The higher dosage of SAP A and SAP B mixes resulted in higher yield stress compared to the rest of the mixes. W-27A exhibited an increase in yield stress from 16.98 Pa (at 20 min) to 46.54 Pa (at 35 min), which is quite similar to W-26A, ranging from 15.19 Pa (at 20 min) to 45.61 Pa (at 35 min). On the other hand, W-39B depicted more yield stress, ranging from 9.19 Pa (at 5 min) to 33.99 Pa (at 35 min), than W-40B, which ranged from 8.60 Pa (at 5 min) to 30.51 Pa (at 35 min).

From Figure 14 and Figure 15, a drastic increase in plastic viscosity and yield stress was observed as the dosage of wet SAP increased in the cement paste with *w/c* ratios of 0.5 and 0.6. The behavior of both wet SAPs with varying dosages followed almost a similar trend for cement paste with *w*/*c* ratios of 0.5 and 0.6. However, the plastic viscosity and yield stress increased when the *w*/*c* ratio was lower, such as for the cement paste with a *w*/*c* ratio of 0.5, compared to the cement paste with a *w*/*c* ratio of 0.6.

Similarly, SAP A had a more significant impact on the plastic viscosity and yield stress of the cement paste with a *w*/*c* ratio of 0.5 than SAP B. The plastic viscosity of W-31A ranged from 1.45 Pa·s to 3.03 Pa·s (30 to 35 min), surpassing that of W-32A, which ranged from 1.50 Pa·s to 2.44 Pa·s. As the dosage of SAP B increased, the plastic viscosity also increased, as shown in Figure 14b. The yield stress of W-32A was higher, ranging from 7.27 Pa to 51.55 Pa over the test duration, whereas for W-44B, the yield stress ranged from 8.29 Pa to 25.96 Pa, as illustrated in Figure 14c,d. 

From Figure 15a,b, both SAP A and B showed a similar trend of increasing plastic viscosity with an increase in dosage over the testing duration. The W-36A mix depicted higher plastic viscosity, ranging from 0.23 Pa·s to 1.30 Pa·s (from 5 min to 35 min), whereas W-48B ranged from 0.31 Pa·s to 1.35 Pa·s. The plastic viscosity for W-35A and W-47B (0.4% SAP) exhibited fluctuations during the test duration. However, there was an overall increase over the test period compared to the reference mix. Specifically, for W-35A, there was a continuous increase in plastic viscosity from 0.22 Pa·s at 5 min to 1.23 Pa·s at 35 min. Similarly, for W-47B, the plastic viscosity increased from 0.23 Pa·s at 5 min to 0.96 Pa·s at 35 min.

From Figure 15c,d, cement paste with 0.5% SAP, i.e., W-36A and W-48B, demonstrated varying yield stress trends throughout the test duration. Specifically, for W-36A, the yield stress increased from 4.89 Pa at 5 min to a peak of 24.91 Pa at 35 min. In contrast, for W-48B, the yield stress also increased over time, starting at 4.64 Pa at 5 min and reaching 21.86 Pa at 35 min. Both W-36A and W-48B exhibited an upward trend in yield stress over the 35 min test period, with W-36A showing a more pronounced increase and finishing with a higher peak value compared to W-48B. The W-47B mix exhibited the highest yield stress compared to the rest of the mixes, ranging from 4.42 Pa to 29.48 Pa throughout the duration.

The *w*/*c* ratio and SAP dosage significantly impact plastic viscosity and yield stress. Plastic viscosity and yield stress were higher in the mixes with a *w*/*c* ratio of 0.4, decreasing as the *w*/*c* ratio increased to 0.5 and 0.6. Mixes with a *w*/*c* ratio of 0.6 showed very low plastic viscosity and yield stress, as they were highly flowy during the test, whereas mixes with a *w*/*c* ratio of 0.4 became very thick over the test duration.

In a study by Dang et al. (2017), the addition of wet SAP in cement-based mixes increased the slump largely compared to the slump of mixes with dry SAP when additional internal curing water was added. However, without the additional internal curing water, the slump decreased severely, and the slump was less affected by the change in particle size of SAP [22]. Similarly, in this study, the addition of wet SAP resulted in a more significant increase in plastic viscosity and yield stress compared to the mixes with dry SAP. This may be due to the addition of presoaked SAP reducing the effective water content, equivalent to reducing water. Without providing additional water, it may lead to an increase in plastic viscosity and yield stress. Additionally, when the dry SAP is mixed with cement, the intricate ion composition in the cement mix prevents the SAP from fully absorbing the water. Consequently, fine cement particles cling to the SAP particles, elevating the friction [23], which could also be the reason for the lower plastic viscosity and yield stress of cement paste with dry SAP compared to cement paste with wet SAP.

**Figure 16:**
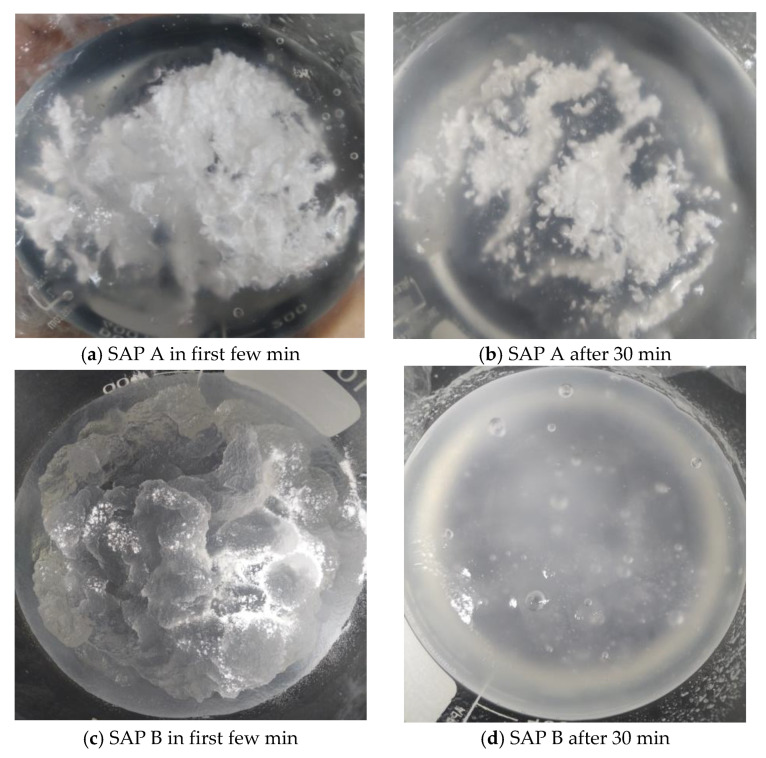
SAP A and SAP B mixed with water for 30 min presoaking.

During wet SAP mixing, SAP A and SAP B were presoaked in water for 30 min; however, the addition of SAP A in water resulted in gel blocking, which was similar to what was observed during the absorption testing. Even after 30 min of presoaking SAP A in water, the particles were not fully swollen, as shown in Figure 16. This phenomenon does not occur in dry SAP mixing because the cement and dry SAP were mixed before adding water. On the other hand, SAP B, which had a larger particle size, was not affected by gel blocking and was fully absorbed during the 30 min. The gel blocking by SAP A was very severe when a higher dosage of SAP and a *w*/*c* ratio of 0.4 were used. In cement paste with wet SAP A mixes, plastic viscosity and yield stress were also affected by gel blocking caused by SAP A.

In order to prevent gel-blocking, it is suggested that dry SAP be used and mixed with cement before adding water. Alternatively, if presoaked SAP is used in the cement paste, using SAP with a particle size above 100 µm is recommended to avoid gel blocking [2,29].

### 3.3. Comparative Analysis and Applications

The SAP used in this study showed higher absorption in pH 7 and pH 11 solutions, which correspond to neutral water and mildly alkaline concrete admixture environments. This indicates that the SAP has good water retention properties despite the alkaline environment.

Based on the results of the absorption capacity in pH 13 and cement filtrate solution, it was observed that the absorption capacity of both SAPs gradually decreased. However, in cement paste, both SAPs exhibited a continuous increase in plastic viscosity and yield stress throughout the test. This suggests that SAPs continuously absorb water in cement paste, demonstrating their ability to retain water in a cementitious environment.

Based on these findings, the polyacrylic-based SAPs used in this study can be effectively employed for internal curing purposes. They can effectively maintain moisture and contribute to promoting hydration in various construction materials and applications where low workability is necessary, such as plastering and grouting. For highly workable cementitious composites, the addition of a superplasticizer can be considered in SAP-modified cementitious materials.

## 4. Conclusions

This paper assesses the absorption capacity of two different sizes of SAP in various pH environments, examining their impact on the rheological properties of cement paste prepared using three different *w*/*c* ratios: 0.4, 0.5, and 0.6, with varying SAP dosages ranging from 0.2 to 0.5% weight of cement. The findings lead to the following conclusions:

1. The absorption capacity of SAP A (avg. size of 28 µm) surpasses that of SAP B (avg. size of 80 µm) across all three pH solutions in both methods, such as the tea-bag and filtration methods. The filtration method depicted a higher absorption capacity than the tea-bag method. Notably, SAP A exhibits lower initial absorption in pH 11 due to its finer size, attributed to the gel-blocking effect.
2. In the tea-bag method, SAP A absorbs more solution in pH 11 (46.65 g/g at 24 h) compared to pH 7 (36.74 g/g at 24 h) and pH 13 (33.20 g/g at 24 h). SAP B displays signs of water release in all instances after 30–60 min. However, in pH 13, a significant increase in absorption capacity is observed, rising from 23.89 g/g at 3 h to 30.10 g/g at 24 h. The absorption capacity of both SAPs decreases as the pH of the solution increases from 11 to 13.
3. Both SAPs demonstrated lower absorption in the cement filtrate solution (pH of 13.73). The absorption by SAP A, using the tea-bag and filtration methods at 1 min, was 22.68 g/g and 48.06 g/g, respectively, then decreased to 2.19 g/g and 26.54 g/g after 24 h. Similarly, for SAP B, the absorption capacity using the tea-bag and filtration methods at 1 min was 29.74 g/g and 46.39 g/g, respectively, which then decreased to 2.31 g/g and 23.55 g/g after 24 h, respectively.
4. The introduction of SAP leads to higher plastic viscosity and yield stress despite the inherent desorption behavior observed in the cement filtrate solution. Both SAPs exhibit increased plastic viscosity and yield stress in mixes with *w*/*c* ratios of 0.4, 0.5, and 0.6, containing varying dosages of dry and wet SAP compared to reference mixes. Furthermore, adding wet SAP resulted in a more significant increase in plastic viscosity and yield stress compared to mixes with dry SAP.
5. Cement pastes with SAP and a *w/c* ratio of 0.4 exhibited higher plastic viscosity and yield stress. As the *w/c* ratio increased to 0.5 and 0.6, the viscosity and yield stress decreased, but they remained higher than the reference mixes without SAP. Mixes containing SAP and a *w/c* ratio of 0.6 demonstrated significantly lower plastic viscosity and yield stress compared to those with a *w*/*c* ratio of 0.4. This indicates that mixes with a *w*/*c* ratio of 0.6 were highly fluid, while those with a *w*/*c* ratio of 0.4 became very thick due to the addition of SAP.
6. Both SAPs in dry form increased the plastic viscosity and yield stress of cement paste with *w*/*c* ratios of 0.4, 0.5, and 0.6. However, when the mix contained 0.2% SAP, the plastic viscosity and yield stress were almost similar to the reference mixes at *w*/*c* ratios of 0.5 and 0.6. This suggests that incorporating 0.2% SAP has a significantly lesser impact on the rheological properties, making it a viable option for practical applications.

The results obtained in this study for cement paste with SAP will further be considered in evaluating the impact of SAP in concrete and mortar, specifically for understanding changes in fresh concrete properties, microstructure analysis, and the impact on mechanical properties.

## Figures and Tables

**Table 1:** Mix proportions of cement paste with different *w*/*c* ratios and dosages of SAPs.

Sample	*w*/*c*	SAP/c (%)	Sample	*w*/*c*	SAP/c (%)	Sample	*w*/*c*	SAP/c (%)
D-1A	0.4	0.2	D-17B	0.5	0.2	W-33A	0.6	0.2
D-2A	0.4	0.3	D-18B	0.5	0.3	W-34A	0.6	0.3
D-3A	0.4	0.4	D-19B	0.5	0.4	W-35A	0.6	0.4
D-4A	0.4	0.5	D-20B	0.5	0.5	W-36A	0.6	0.5
D-5A	0.5	0.2	D-21B	0.6	0.2	W-37B	0.4	0.2
D-6A	0.5	0.3	D-22B	0.6	0.3	W-38B	0.4	0.3
D-7A	0.5	0.4	D-23B	0.6	0.4	W-39B	0.4	0.4
D-8A	0.5	0.5	D-24B	0.6	0.5	W-40B	0.4	0.5
D-9A	0.6	0.2	W-25A	0.4	0.2	W-41B	0.5	0.2
D-10A	0.6	0.3	W-26A	0.4	0.3	W-42B	0.5	0.3
D-11A	0.6	0.4	W-27A	0.4	0.4	W-43B	0.5	0.4
D-12A	0.6	0.5	W-28A	0.4	0.5	W-44B	0.5	0.5
D-13B	0.4	0.2	W-29A	0.5	0.2	W-45B	0.6	0.2
D-14B	0.4	0.3	W-30A	0.5	0.3	W-46B	0.6	0.3
D-15B	0.4	0.4	W-31A	0.5	0.4	W-47B	0.6	0.4
D-16B	0.4	0.5	W-32A	0.5	0.5	W-48B	0.6	0.5

## Data Availability

The original contributions presented in the study are included in the article, further inquiries can be directed to the corresponding author.
